# Identification Hub Genes in Colorectal Cancer by Integrating Weighted Gene Co-Expression Network Analysis and Clinical Validation *in vivo* and *vitro*

**DOI:** 10.3389/fonc.2020.00638

**Published:** 2020-04-30

**Authors:** Yihang Yuan, Ji Chen, Jue Wang, Ming Xu, Yunpeng Zhang, Peng Sun, Leilei Liang

**Affiliations:** Department of General Surgery, Tongren Hospital, Shanghai Jiao Tong University School of Medicine, Shanghai, China

**Keywords:** colorectal cancer, hub gene, weighted gene, co-expression network analysis, clinical validation

## Abstract

Colorectal cancer (CRC) is the third leading cause of death in the world. However, the key roles of most molecules in CRC remain unclear. This study aimed to identify key modules and hub genes associated with the progression of CRC. The data of the patients with CRC were obtained from the Gene Expression Omnibus (GEO) database and assessed by weighted gene co-expression network analysis (WGCNA), Gene Ontology (GO) and Kyoto Encyclopedia of Genes and Genomes (KEGG) enrichment analyses performed in R. by WGCNA, several hub genes that regulate the mechanism of tumorigenesis in CRC were identified, which were associated with clinical traits. Next, we screened hub genes related to the progression of CRC authenticated by The Cancer Genome Atlas (TCGA) and Oncomine databases. Three hub genes (HCLS1, EVI2B, and CD48) were identified, and survival analysis was further performed. Moreover, the results of qPCR and immunohistochemistry staining revealed that HCLS1, EVI2B, and CD48 are tumor suppressor genes. Further, the functional study verified that over-expression of HCLS1, EVI2B, and CD48 can reduce the proliferation, migration, and invasion ability of CRC cells and significantly suppress CRC tumor growth *in vivo*. In summary, we identified three hub genes that were associated with the progression of CRC that can be applied in treatment.

## Introduction

Colorectal cancer (CRC) is the most common types of gastrointestinal tumors and deadly cancer globally (1). Currently, adenomas can be surgically removed, and the prognosis is favorable in the early stages. However, once cancer metastasizes to organs, the survival rates of CRC patients significantly decline (2). Therefore, it is important to explore the pathogenesis of CRC, which for developing optimal therapeutic strategies.

In recent years, new generation of sequencing technology has achieved breakthrough development, and an increasing number of scientists have recognized the crucial role of sequencing in life science research (3). The high-throughput platform is considered to be the most useful instrument for analyzing tumor genes (4) and has good application prospects in the search for diagnostic and therapeutic markers for tumors (5). However, how to translate the microarray information into a better understanding of biology through traditional differential expression analysis remains a major challenge (6). Weighted gene co-expression network analysis (WGCNA) can be implemented as a data exploratory tool or as gene screening method to explore candidate biomarkers and therapeutic targets (7). For example, WGCNA can be applied in the description of correlation structure between gene expression profiles (8), data of genetic marker (9), data of proteomics (10), and other datas of high-dimensional in various biological processes (11). WGCNA can be helpful for processing of comparing differentially expressed genes and determining the interactions among genes in different co-expression modules (12).

In this present study, we first downloaded the GSE17538 and GSE29623 datasets from the Gene Expression Omnibus (GEO) database, which included 303 CRC samples and matched para-cancer normal samples, and then screened the mRNA expression profiles. Then, these data were analyzed by WGCNA, Gene Ontology (GO), and Kyoto Encyclopedia of Genes and Genomes (KEGG). Finally, we screened three hub genes (HCLS1, EVI2B, and CD48) that may be involved in the prognosis of CRC patients. Based on profiles of the hub gene expression in the test set, we analyzed the expression of mRNAs by qPCR and immunohistochemistry staining in colon cancer samples. Furthermore, the functional study confirmed that the overexpression of HCLS1, EVI2B, and CD48 can reduce the proliferation, migration and invasion ability of CRC cells *in vitro* and significantly suppress CRC tumor growth *in vivo*. Three hub genes may be very beneficial for assessing the progression of CRC and can be applied in treatment.

## Materials and Methods

### Data Processing

We downloaded the GSE17538 (238 cases) and GSE29623 (65 cases) datasets from the GEO database. We annotated probes using the Affymetrix Human Genome U133 Plus 2.0 Array platform, and clinical information was used for WGCNA. Batch analysis was used to correct batch differences between the two datasets. We then selected the top 25% most variant genes to construct the co-expression network with the WGCNA package in R. The power value was calculated by the pickSoftThreshold function, and the network module was segmented using a dynamic tree cutting algorithm.

### Module Determination

The correlation between modules and clinical features, including disease, sex, time to live, and doubling time for WGCNA, were assessed by Pearson correlation coefficients. Module significance was defined as the average GS of all genes in a module, which was used for screening key modules.

### Gene Enrichment Analysis

We performed GO and KEGG pathway analysis of the clinically significant module by R software. We used “adjusted *p* < 0.05” as the cut-off criteria to screen out the enriched GO terms and KEGG pathways.

### Identification of Hub Genes

In weighted co-expression networks, “CRC-related mRNAs” are referred to as nodes. Genes closely linked to the nodes in the modules are considered to have important functions and regarded as hub genes. Hub genes often have important biological functions and are closely related to other nodes of the module. We used Cytoscape to draw the network diagram of key modules and to screen out genes with degrees ranking in the top 30 in the module network. To verify the reliability of the hub genes, colon cancer data in the The Cancer Genome Atlas (TCGA) database was used for further validation. The Kaplan–Meier plotter was used for survival analysis on the data from the TCGA database.

### Gene Set Enrichment Analysis

We separated CRC samples into high-expression and low-expression groups using gene set enrichment analysis (GSEA) version 2.2.1 software. We selected c2.cp.kegg.v6.2.symbols.gmt (Molecular Signature Database version 6.2) as the reference gene set. Then, enrichment analysis was carried out by default weighted enrichment statistics, and the number of random combinations was set to 1,000.

### Sample Collection

CRC and adjacent tissues were collected from 30 patients (all participants were older than 16 years), immediately placed in liquid nitrogen, and preserved at −80°C. None of the CRC patients received preoperative anti-tumor therapies. Patients and their families in this study have been fully informed and informed consent was obtained from the participants. This study was approved by the Ethics Committee of Shanghai Tongren Hospital.

### Cell Culture and Transfection

Human normal colorectal epithelial cell line (NCM460), CRC cell line (including SW480, SW620, CoLo205, and Lovo cells) and umbilical vein endothelial cells (HUVECs) were obtained from the Shanghai Cell Bank of the Chinese Academy of Sciences (Shanghai, China). All of them were cultured in 90% DMEM (Gibco) supplemented with antibiotics (1 × penicillin/streptomycin 100 U/ml, Gibco) and 10% heat-inactivated fetal bovine serum (FBS; Gibco, Grand Island, NY, USA). The cells were incubated at 37°Cin a humidified and 5% CO_2_ incubator.

Full-length pcDNA 3.3-HCLS1, pcDNA 3.3-EVI2B, pcDNA 3.3-CD48, and pcDNA 3.3-NC vectors were purchased from RiboBio (Guangzhou, China), and transfected into CoLo cells using FuGENE HD Transfection Reagent (Promega, USA) as previously described (13). The HCLS1, EVI2B, or CD48 transfection efficiency was ~85, 81, or 80%, respectively. Forty-eight hours post-infection, the cells were collected and processed for various assays.

### RNA Isolation and PCR Analysis

Total RNA from the CRC tissues and cells was extracted by TRIzol reagent (Invitrogen, Thermo Scientific, Shanghai, China), and RNA was reverse transcribed into cDNA with Reverse Transcription Kit (QIAGEN, USA). qPCR analyses were quantified with SYBR-Green (Takara, Japan), and the gene expression were normalized to GAPDH.

### Immunohistochemical Staining

Paraffin-embedded tissues were immunostained for HCLS1, EVI2B, and CD48 proteins. The slides were dried at 60°C, dewaxed with methanol and rehydrated with alcohol. Then, the slides were immersed in 3% hydrogen peroxide and labeled with antibodies overnight. Anti-HCLS1 (1:200), anti-EVI2B (1:200), and anti-CD48 (1:200) were purchased from Invitrogen (Shanghai, China). The protein expression analysis by Image-Pro Plus 6.0 Software.

### Cell Proliferation Assay

Cell proliferation was evaluated by cell counting kit-8 (CCK-8) (Beyotime, Shanghai, China). Human CRC cells (1 × 10^5^) were seeded in a 96-well-plate of each well and cultured for 24, 48, and 72 h. The absorbance value at 450 nm was read via a Varioskan Flash (Bio-Rad,USA).

### Colony Formation Assay

For the colony formation assay, 1 × 10^3^ cells were seeded in 6-well-plates. The cells were thoroughly resuspended and then incubated for 7 days in culture medium with 10% FBS. A single colony were counted for clusters containing ≥30 cells.

### Cell Migration and Invasion Assays

The invasion and migration abilities of CRC cells were evaluated by a Transwell assay (8 μ m pore size, Corning, USA). Cells were treated with HCLS1-pcDNA 3.3, EVI2B-pcDNA 3.3, and CD48-pcDNA 3.3 for 48 h and then harvested. A total of 5 × 10^4^ cells in serum-free medium were placed into the upper chamber of an insert of the Transwell assay, while medium supplemented with 10% FBS was added to the lower chamber for chemo-attraction. Transwell membrane filters were used for the migration assays, and Transwell membrane filters coated with Matrigel (Corning, NY, USA) were used for the invasion assays. After incubation at 37°C for 24 h, the cells were stained with methanol and 0.1% crystal violet, imaged, and counted using an Olympus microscope (Tokyo, Japan).

### Tumorigenicity Assay *in vivo*

We used male nude mice (6 weeks of age) obtained from the Animal Facility of Shanghai Jiao Tong University School of Medicine; the mice were fed sterilized food and water. All of the animal experiments were approved by the responsible governmental animal ethics committee and complied with the ARRIVE guidelines and the Ethics Committee of Shanghai Tongren Hospital. A cell suspension of CoLo205 cells (2 × 10^6^ in 100 μ l) labeled with luciferase was injected into the submucosa of the terminal cecum wall of the nude mice. The pcDNA 3.3-HCLS1, pcDNA 3.3-EVI2B, pcDNA 3.3-CD48, and pcDNA 3.3-NC were optimized by RiboBio (Guangzhou, China). We make the transfection reagent and plasmid into complex and the ratio of EntransterTM-*in vivo* (Engreen, Beijing, China) DNA is 2:1 and the dosage of pcDNA 3.3 is 3 mg/kg, according to the instructions. We injected the complex into the tail vein of mice, twice a week for 4 weeks. The mice were observed for 4 weeks by the Caliper IVIS Lumina II System.

### Statistical Analysis

The student t test was used to examine differences between groups. *P* < 0.05 was statistically significant. All calculations were performed by SPSS version 13.0.

## Results

### Differentially Expressed mRNA Analysis

**Figure 1:**
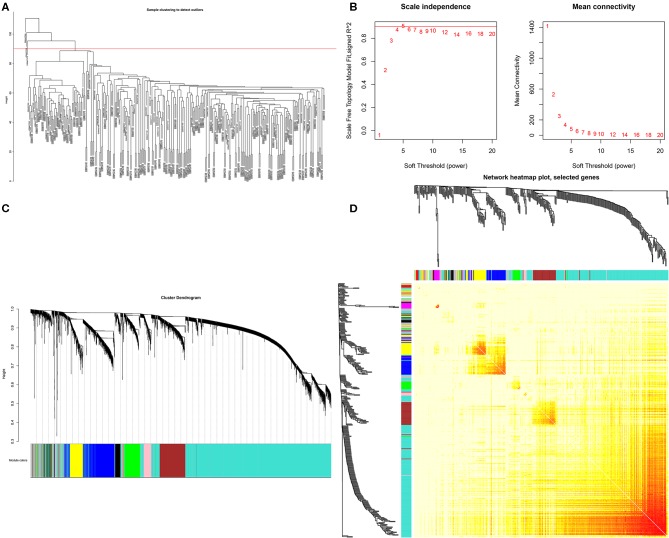
Network visualization plots. **(A)** Based on the hierarchical clustering graphs of GSE17538 and GSE29623, three outliers were removed. **(B)** The appropriate soft threshold power = 5 was selected. **(C)** Gene cluster tree. Based on the adjacency-based dissimilarity of the hierarchical clustering gene clustering chart, the tree diagram below the color was established by the dynamic tree cutting method recognition module. **(D)** Interaction analysis of co-expressed genes. The different colors of the horizontal and vertical axes represent different modules. The yellow brightness in the middle indicates the degree of correlation between different modules.

**Figure 2:**
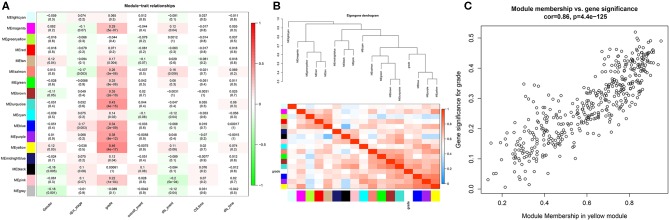
Heat map of the correlation between the module and the clinical features of the disease. **(A)** The numbers above represent correlations, followed by *P*-values; red indicates a positive correlation, and green indicates a negative correlation. **(B)** Module and clinical hierarchy clustering and heat maps for summarizing the modules generated in the WGCNA. **(C)** Yellow module MM and GS relationship scatter plot.

In this study, we downloaded the GSE17538 (238 cases) and GSE29623 (65 cases) datasets from the GEO database. We annotated probes using the Affymetrix Human Genome U133 Plus 2.0 Array platform, and clinical information was used for WGCNA. The top 25% most variant genes in the corrected chip data were used for clustering analysis by the WGCNA package (Figure 1A). To ensure the reliability of the network structure, three outliers are deleted, and 300 samples remained (Figure 1B). The gene expression data of the top 25% most variant genes were screened to construct a weighted gene co-expression network. The power value of 5 (Figure 1C) was chosen, and 14 modules were generated, with gray modules representing non-co-expressed genes. Afterwards, we analyzed the interactive relations among the 14 modules, drew the heat map of network, and showed the relative independence of each module (Figure 1D). As showed in (Figure 2A), the yellow module was positively correlated with grade and has the highest correlation compared to other modules. Furthermore, we exigencies the eigengenes of modules and clustered them on the basis of their correlation with grade. The yellow module was closely related to grade (Figure 2B). Heat maps based on adjacencies also showed similar results. (Figure 2C) illustrates the relationship between the yellow module and genetic signification.

### GO Functional and KEGG Pathway Analyses of mRNAs

**Figure 3:**
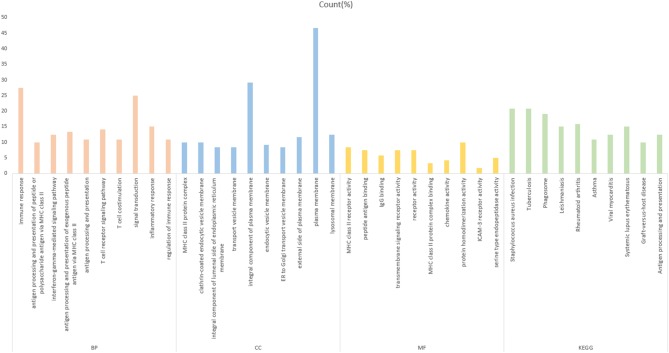
Gene Ontology analysis of the yellow module genes. The abscissa is the percentage of enriched gene entries in the total number of genes in this item, and the orange, blue, yellow, and green colors represent the BP-, MF-, CC-, and KEGG-enriched entries, respectively. The entries enriched by each item are arranged from left to right in the order of the *P*-value from small to large, with all *P* < 0.05.

To further investigate the function of the screened mRNAs in the progression of CRC, GO functional, and KEGG pathway enrichment analyses were performed on the yellow module by the R package clusterProfiler. The results showed that these mRNAs were mainly enriched in three categories, including the biological process (BP), cellular component (CC), and molecular function (MF) categories. The KEGG pathway results showed that these mRNAs were mainly enriched in CRC-related pathways (Figure 3, Supplementary Table 1). The enrichment analysis results are closely related to colon cancer, indicating the correctness of our analysis results.

### Hub Gene Identification and Validation

**Figure 4:**
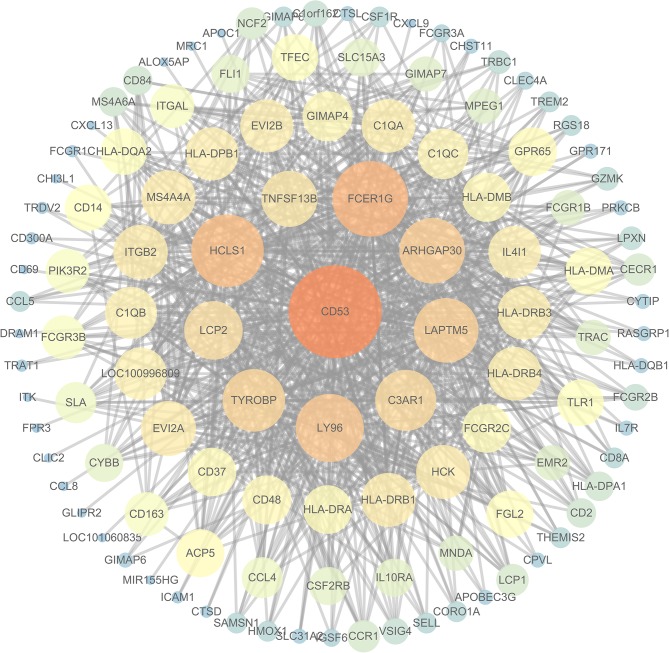
The interaction network diagram of all the nodes in the yellow module: the large orange node is the node with a high degree, and the small blue node is the node with a low degree.

**Figure 5:**
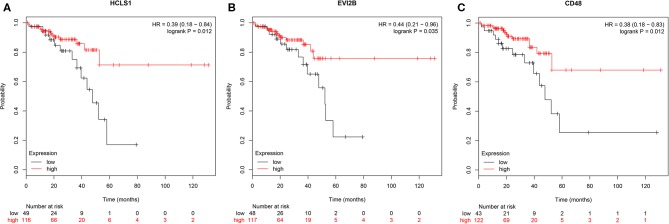
Survival analysis of hub genes. **(A)** HCLS1, **(B)** EVI2B, and **(C)** CD48 were significantly associated with OS. *P* < 0.05 was regarded as statistically significant.

**Figure 6:**
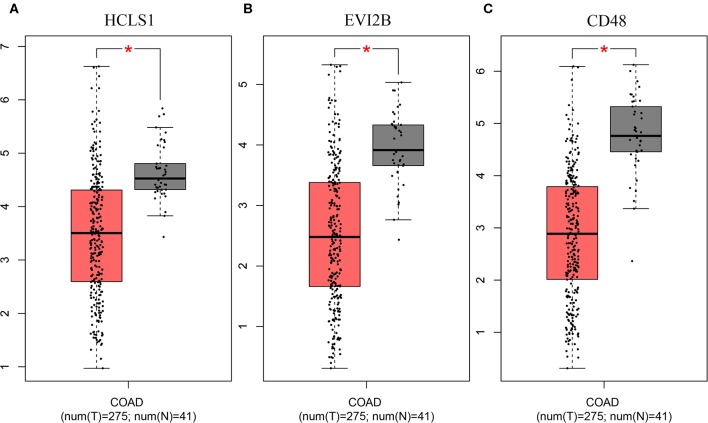
Validation of the hub genes in the TCGA database. The hub genes were verified based on TCGA-COAD data. **(A)** HCLS1 expression was downregulated in colon cancer tissues. **(B)** EVI2B was downregulated in colon cancer tissues. **(C)** CD48 was downregulated in colon cancer tissues. *P < 0.05.

To further investigate the interrelationship among the risk genes associated with CRC, a PPI network was constructed with the mRNAs found above. The yellow module was imported into Cytoscape for topological analysis. The topological parameters of all the nodes in the module network were calculated (Supplementary Table 2), and the top 30 nodes with the highest degree in each module were selected to draw the network diagram (Figure 4) as the candidate key nodes for further analysis. Furthermore, the relationship between the screened mRNAs and the overall survival (OS) rate of CRC patients was analyzed. The results showed that three hub mRNAs (HCLS1, EVI2B, and CD48) were significantly negatively related to the prognosis of patients with CRC (*P* < 0.05; Figure 5). Moreover, we confirmed the expression patterns of these three mRNAs in a colon cancer dataset from the TCGA database. As showed in Figure 6, three hub mRNAs (HCLS1, EVI2B, and CD48) were expressed at low levels in CRC samples relative to that in para-cancer normal samples. Our results demonstrated that the three hub mRNAs we found might be associated with the risk of CRC.

### Gene Set Enrichment Analysis

**Figure 7:**
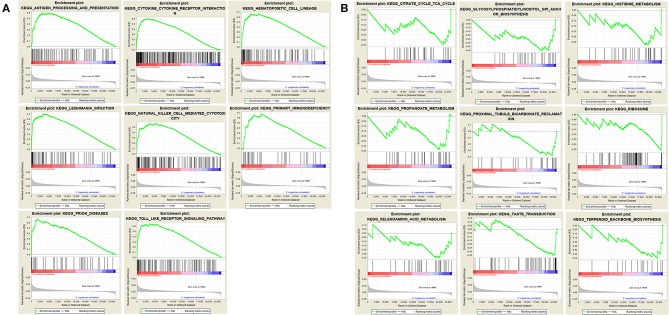
Gene set enrichment analysis. **(A)** The top 10 enriched entries of the three hub gene high-expression group. **(B)** The top 10 enriched entries of the three hub gene low-expression group.

We performed gene set enrichment analysis (GSEA) to further understand the functions of these hub genes. GSEA showed that there were 8 entries intersecting in the first 10 entries enriched by the high expression group of each hub gene, as showed in Figure 7A. There were 9 entries intersecting in the first 10 entries enriched by the low expression group of each hub gene, as showed in Figure 7B.

### Expression of the Hub Genes in CRC

**Figure 8:**
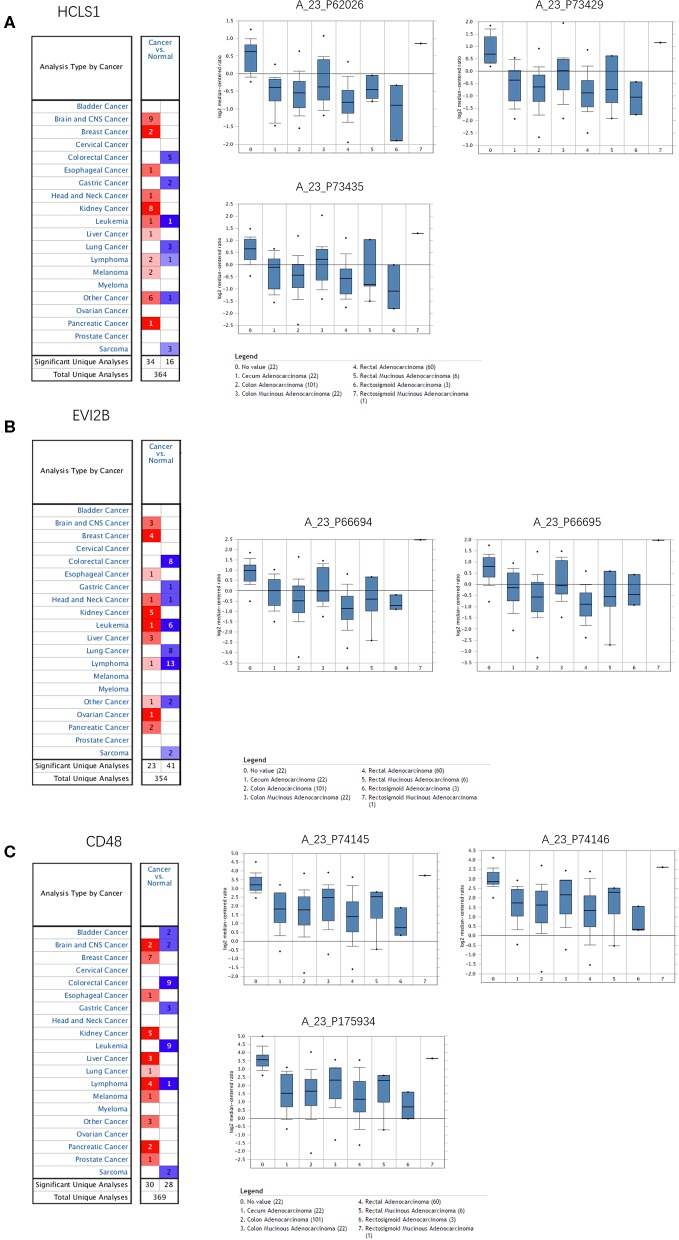
Expression of hub genes in the Oncomine database. **(A)** HCLS1 was downregulated in colon cancer tissues compared with normal tissues in the TCGA dataset (A_23_P62026, A_23_P73429, and A_23_P73435). **(B)** EVI2B was downregulated in colon cancer tissues compared with normal tissues in the TCGA dataset (A_23_P66694 and A_23_P66695). **(C)** CD48 was downregulated in colon cancer tissues compared to normal tissues in the TCGA dataset (A_23_P74145, A_23_P74146, and A_23_P175934).

**Figure 9:**
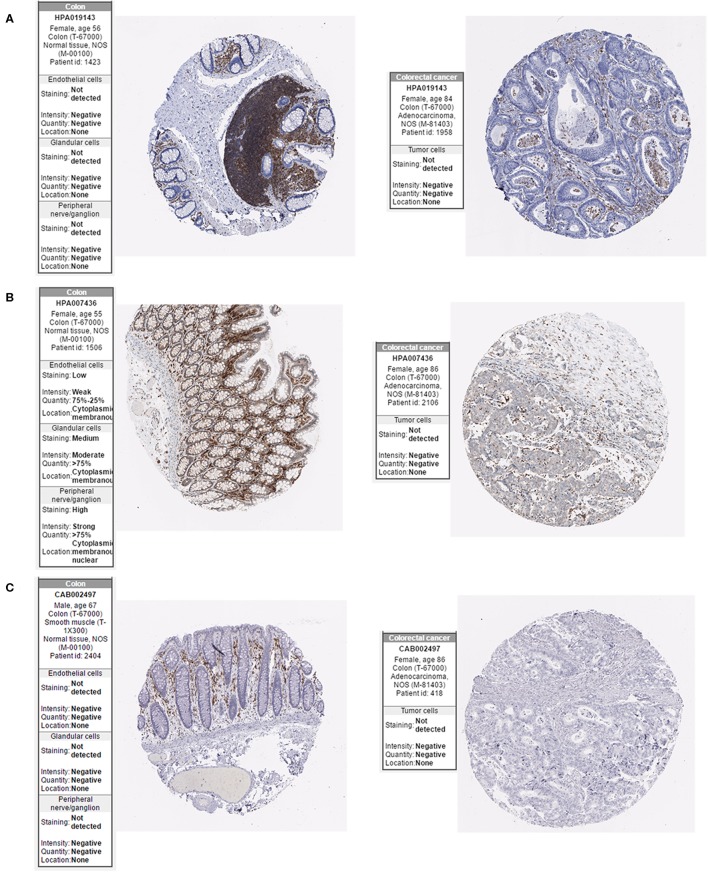
Expression of hub genes in the Human Protein Atlas database. **(A)** HCLS1 expression was downregulated in colon cancer tissues. **(B)** EVI2B was downregulated in colon cancer tissues. **(C)** CD48 was downregulated in colon cancer tissues.

The Oncomine database was used to retrieve the expression of the hub genes in different types of cancer. Comparing the expression of the hub genes in colon cancer in the database, the hub genes showed low expression in almost all types of colon cancer (Figure 8). Furthermore, we observed the expression of the hub genes in CRC using the Human Protein Atlas database, and the results showed that the expression of the hub genes (HCLS1, EVI2B, and CD48) in paracancerous tissues was significantly higher than that in CRC tissues according to immunohistochemistry (Figure 9).

### Hub Gene Expression Is Downregulated in CRC Tissues and Cell Lines

**Figure 10:**
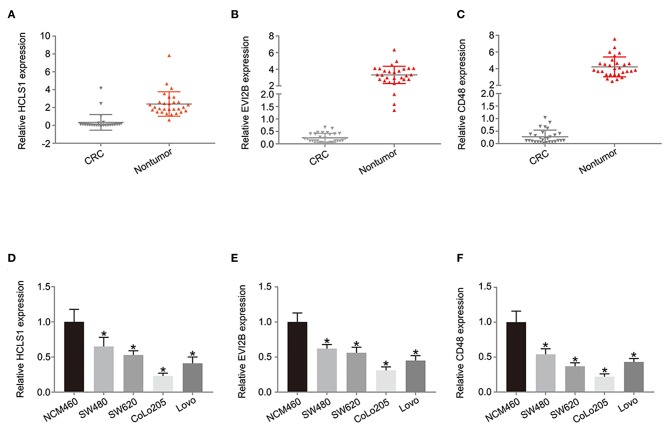
The expression of the hub genes is downregulated in CRC. According to the qPCR results, HCLS1 **(A,D)**, EVI2B **(B,E)**, and CD48 **(C,F)** are downregulated in CRC tissues and cells. *P < 0.05.

**Figure 11:**
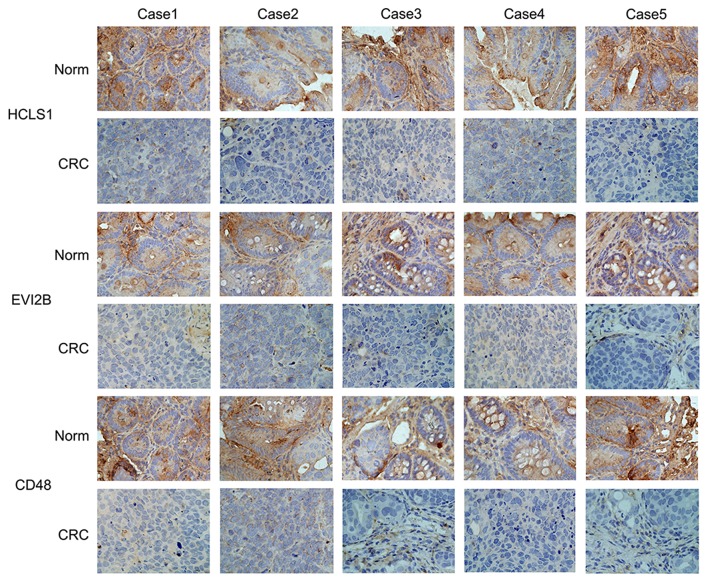
The expression of the hub genes is downregulated in CRC. Immunostaining demonstrated that HCLS1, EVI2B, and CD48 were downregulated in CRC tissues compared with normal tissues.

Next, we examined the expression of the hub genes (HCLS1, EVI2B, and CD48) by qPCR in 30 pairs of clinical samples from CRC patients. The qPCR results shown that hub genes were expressed at low levels in CRC tissues (Figures 10A–C). Correspondingly, immunostaining analyses of the hub genes (HCLS1, EVI2B, and CD48) were performed in the cancerous and paracancerous tissues, and immunostaining demonstrated that the expression of the hub genes (HCLS1, EVI2B, and CD48) was high in the paracancerous tissue (Figure 11). Furthermore, the expression of the hub genes (HCLS1, EVI2B, and CD48) was increased in NCM460 (normal colon) cells compared with that in CoLo205 (highly invasive) cells (Figures 10D–F). The qPCR and immunostaining results were consistent with the bioinformatics analysis results. Next, according to the PCR results, we selected CoLo205 cells for gene function exploration.

### Hub Genes Regulate the Proliferation, Migration, and Invasion Ability of CRC Cells

**Figure 12:**
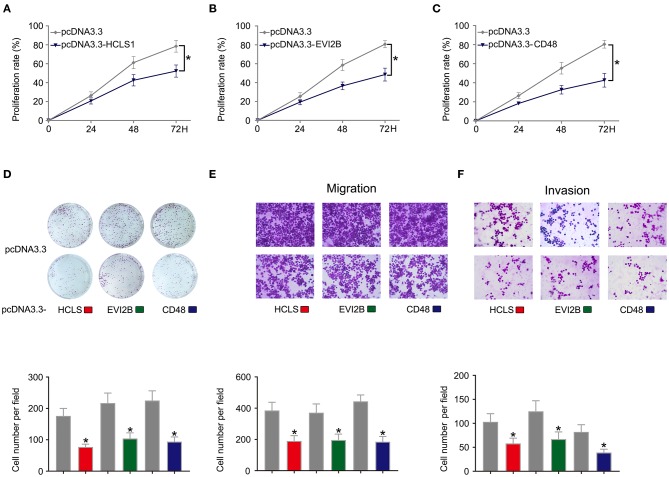
Hub genes regulate the proliferation, migration, invasion, and angiogenic ability of CRC cells. HCLS1, EVI2B, and CD48 inhibited the proliferation of CRC cells according to a CCK-8 assay **(A–C)** and colony formation assay **(D)**. HCLS1, EVI2B, and CD48 can inhibit the development of CRC via weakening the migration and invasion capacity of CRC cells. **(E,F)** HCLS1, EVI2B, and CD48 can inhibit the development of CRC via weakening the migration and invasion capacity of CRC cells. *P < 0.05.

We investigated the role of HCLS1, EVI2B, and CD48 in proliferation by colony formation and CCK-8 assays. The CCK-8 (Figures 12A–C) and colony formation assays (Figure 12D) showed that the overexpression of HCLS1, EVI2B, and CD48 reduced cell proliferation. To examine the effect of HCLS1, EVI2B, and CD48 on cell invasion and migration, transwell assays were performed. The results showed that after the overexpression of HCLS1, EVI2B, and CD48, the cell migration efficiency of CoLo205 cells was suppressed compared with control group (Figures 12E,F). These data suggest that HCLS1, EVI2B, and CD48 can inhibit the proliferation, migration, and invasion of CRC cells.

### Hub Genes Treat CRC *in vivo*

**Figure 13:**
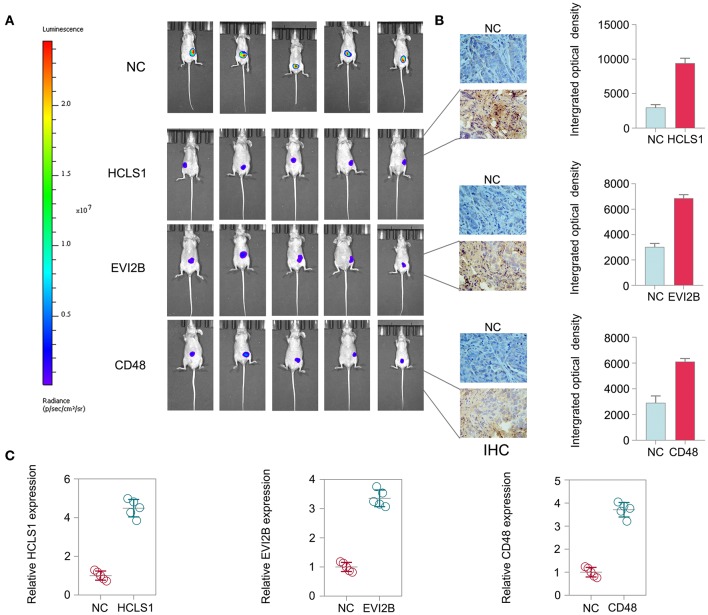
Hub gene treatment in CRC *in vivo*. **(A)** The data indicate that pcDNA 3.3-HCLS1, pcDNA 3.3-EVI2B, or pcDNA 3.3-CD48 significantly suppressed colorectal cancer tumor growth relative to the pcDNA 3.3-NC groups. **(B)** The immunostaining revealed that HCLS1, EVI2B, or CD48 proteins expression were higher in pcDNA 3.3-HCLS1, pcDNA 3.3-EVI2B, or pcDNA 3.3-CD48 compared with pcDNA 3.3-NC group in xenografted tumor. **(C)** The qPCR results showed that the expression of HCLS1, EVI2B, or CD48 was increased in pcDNA 3.3-HCLS1, pcDNA 3.3-EVI2B, or pcDNA 3.3-CD48 compared with pcDNA 3.3-NC group.

To further explore the therapeutic effect of pcDNA 3.3-HCLS1, pcDNA 3.3-EVI2B, and pcDNA 3.3-CD48 *in vivo*, we constructed a CRC nude mouse model. The mice were divided into a control group and a treatment group. The mice were then administered pcDNA 3.3-HCLS1, pcDNA 3.3-EVI2B, pcDNA 3.3-CD48, or pcDNA 3.3-NC by injection twice a week for 4 weeks. The data indicate that pcDNA 3.3-HCLS1, pcDNA 3.3-EVI2B, or pcDNA 3.3-CD48 significantly suppressed tumor growth relative to that of the pcDNA 3.3-NC group (Figure 13A). The immunostaining revealed that HCLS1, EVI2B, or CD48 proteins expression were higher in pcDNA 3.3-HCLS1, pcDNA 3.3-EVI2B, or pcDNA 3.3-CD48 compared with pcDNA 3.3-NC group in xenografted tumor (Figure 13B). The qPCR results showed that the expression of HCLS1, EVI2B, or CD48 was increased in pcDNA 3.3-HCLS1, pcDNA 3.3-EVI2B, or pcDNA 3.3-CD48 compared with pcDNA 3.3-NC group (Figure 13C). Taken together, these data suggest that HCLS1, EVI2B, or CD48 are involved in tumorigenesis and that the overexpression of these hub genes receded CRC cell proliferation *in vivo*.

## Discussion

CRC remains the most common types of gastrointestinal tumors and is the cause of a substantial number of deaths in China (14). Effective biomarkers and diagnostic techniques for the early detection of CRC will improve the survival rate of patients (15), and a better understanding of the molecular mechanism associated with CRC progression is essential for establishing effective treatment methods (16).

The high-throughput platform and gene microarray analysis provide tools for investigating tumor markers (17, 18). However, translating the information into a better biological understanding by conventional differential expression analysis remains a major challenge (19). In this study, we first downloaded the GSE17538 and GSE29623 datasets from the GEO database, which included 303 CRC samples, and then screened the mRNA expression profiles. Then, these data were assessed by WGCNA, GO, and KEGG enrichment analyses performed by R software. Moreover, we confirmed the expression patterns of these three mRNAs in a colon cancer dataset from the TCGA database. The hub genes were expressed at low levels in CRC samples relative to that in normal tissues. The results in the Oncomine database and Human Protein Atlas database showed similar findings with respect to gene expression. Furthermore, the results of the overall survival (OS) analysis showed that the three hub genes were significantly negatively associated with the prognosis of patients with CRC (*P* < 0.05). Thus, the three screened hub genes (HCLS1, EVI2B, and CD48) may be involved in the prognosis of CRC patients.

mRNAs play an important regulatory role in numerous biological processes, tumorigenesis, and development (20). Recent publications have revealed that SNX10 (21) and SMAD4 (22) act as tumor suppressors in CRC and could be potential therapeutic targets. CP1 (23) and ANG2 (24) were associated with promoting tumor progression and metastasis and may be useful targets for cancer therapy. In the enrichment analysis results, immune response, inflammatory response, regulation of immune response, T cell receptor signaling pathway, T cell costimulation, antigen processing, and presentation of peptides or polysaccharide antigen via MHC class II, and antigen processing and presentation of exogenous peptides or Polysaccharide antigen via MHC class II and other biological processes are closely related to the occurrence and development of colon cancer. Carter, Marty, and their team found a clear correlation between the composition of the human MHC-II gene and the mutated gene in the human cancer (25). The cancer antigen presented by MHC-II will lead T cells to eliminate these cancer cells earlier. Previous studies showed that the positive expression rate of MHC-II in colon cancer was significantly lower than that in adjacent tissues (*P* < 0.01) (26). In recent years, it has been found that a large number of costimulatory molecules have the ability to affect T cell activation, proliferation, survival, and secretion of cytokines. By manipulating costimulatory molecules to increase the number and activity of tumor antigen-specific T cells and inhibit tumor growth. The expression of T-cell costimulatory molecules in colon cancer was significantly higher than that in the control group (27). In the results of GSEA enrichment analysis, the items such as anti-processing and presentation, toll like receptor signaling pathway are related to the progress of colon cancer. Previous studies have shown that toll like receptor (TLR) plays an important role in nonspecific immunity of tumors. TLR, as an important human immune receptor, can recognize a wide range of exogenous pathogens and endogenous ligands from the host itself. In the process of tumorigenesis and development, TLR produces receptors that can be recognized by TLR, and at the same time, through TLR signal transmission, it can stimulate anti-tumor immune response (28). Therefore, TLR plays an important role in tumor immune monitoring. In this study, the topological parameters of all the nodes in the module network were calculated, and the top 30 nodes with the highest degree in each module were selected to draw the network diagram as the candidate key nodes for further analysis. Furthermore, the relationship between the screened mRNAs and the overall survival (OS) rate of CRC patients was analyzed. The results showed that three hub mRNAs (HCLS1, EVI2B, and CD48) were significantly negatively associated with the prognosis of patients with CRC (*P* < 0.05). However, the biological consequences of HCLS1, EVI2B, and CD48 dysregulation in CRC progression have not yet been reported and we screened HCLS1, EVI2B, and CD48, which may be associated with CRC progression. Among them, studies have shown that HCLS1 might be a potential therapeutic target gene of chronic lymphocytic leukemia (29). EVI2B enhanced the migration of NF1-associated malignant peripheral nerve sheath tumors (30). CD48 plays a vital role as an environmental sensor for regulating stem cells and inhibiting tumor development (31). In our study, we analyzed the expression of HCLS1, EVI2B, and CD48 by qPCR and immunohistochemistry staining in CRC samples. The results showed that the hub genes were significantly downregulated in CRC, which is consistent with the above results. Further, our data from the functional study confirmed that the overexpression of HCLS1, EVI2B, and CD48 can reduce the ability of proliferation, migration, and invasion in CRC cells and significantly suppress CRC tumor growth *in vivo*. Collectively, the genes found in our study might play an important role in the development of CRC. Previous evidence has indicated that these hub genes play regulatory roles with other proteins. HCLS1 associated with the cellular migration of proteins (32). EVI2B is involved in the T-cell-mediated system-wide modulation of protein-protein interactions (33), and CD48 is related to extracellular protein interactions (34). However, we did not consider the proteins associated with the hub genes. Therefore, further study is needed to clarify the underlying molecular mechanisms.

In conclusion, we identified three hub genes, HCLS1, EVI2B, and CD48, that are associated with the prognosis of CRC using systems biology-based co-expression analysis. Our data from the functional study confirmed that HCLS1, EVI2B, or CD48 suppresses CRC progression by inhibiting the proliferation, migration, and invasion capacity of CRC cells as well as tumor growth *in vivo*. These results suggested that HCLS1, EVI2B, and CD48 could be biomarkers for drug targets of CRC patients. Further study is needed to clarify the underlying molecular mechanisms.

## Data Availability Statement

Publicly available datasets were analyzed in this study, these can be found in the NCBI Gene Expression Omnibus (GSE17538, GSE29623).

## Ethics Statement

All of the animal experiments were approved by the Ethics Committee of Shanghai Tongren Hospital.

## Author Contributions

LL and PS conceived this study. YY and YZ performed the experiments. JC, MX, and JW prepared the manuscript. All authors approved the final version of the manuscript.

## Conflict of Interest

The authors declare that the research was conducted in the absence of any commercial or financial relationships that could be construed as a potential conflict of interest.
